# A Drosophila Reporter for the Translational Activation of ATF4 Marks Stressed Cells during Development

**DOI:** 10.1371/journal.pone.0126795

**Published:** 2015-05-15

**Authors:** Kwonyoon Kang, Hyung Don Ryoo, Jung-Eun Park, Jee-Hyun Yoon, Min-Ji Kang

**Affiliations:** 1 Department of Biomedical Sciences, University of Ulsan College of Medicine, Seoul, Republic of Korea; 2 Department of Cell Biology, New York University School of Medicine, New York, New York, United States of America; University of British Columbia, CANADA

## Abstract

Eukaryotic cells have evolved signaling pathways that help to restore cellular homeostasis in response to various physiological or pathological conditions. ATF4 is a transcription factor whose mRNA translation is stimulated in response to stress-activated eIF2alpha kinases. Established conditions that activate eIF2alpha phosphorylation and ATF4 translation include excessive stress in the endoplasmic reticulum (ER) and amino acid deprivation. ATF4 is activated through a unique translational activation mechanism that involves multiple upstream open reading frames (uORFs) in the 5’-untranslated region (UTR), which is conserved from yeast to mammals. Taking advantage of this, we developed a translational activation reporter of ATF4 in *Drosophila*, in which the dsRed reporter coding sequence was placed downstream of the *Drosophila* ATF4 5’ UTR. This reporter remained inactive in most tissues under normal conditions, but showed dsRed expression when starved, or when challenged with conditions that imposed ER stress. In normally developing flies, a small number of cell types showed reporter expression even without exogenous stress, which included the salivary gland, gut, the male reproductive organ, and the photoreceptor cells, suggestive of inherent stress during the normal development of these cell types. These results establish a new tool to study ATF4-mediated stress response in *Drosophila* development and disease.

## Introduction

The endoplasmic reticulum (ER) is a cellular organelle in which secretory and membrane proteins are synthesized, and folded. The function of the ER is often perturbed when the level of protein expression exceeds the folding capacity of ER chaperones. In order to overcome such stress, adaptive signaling pathway known as the unfolded protein response (UPR) is activated. Unresolved ER stress or defective UPR is linked with a number of diseases including certain types of neurodegenerative diseases [1], bipolar disorder [2], atherosclerosis [3], ischemia [4] and metabolic diseases [5, 6]. In mammalian cells, three principal arms of UPR have been identified. One of those signaling arms is mediated by PKR-like ER kinase (PERK) [7]. This transmembrane kinase is activated upon excessive stress in the ER, and ultimately activates a downstream transcription factor, ATF4, a member of the leucine-zipper ATF/CREB family of DNA binding proteins [8]. ATF4 is also activated downstream of other stress-activated kinases. For example, in response to amino acid deprivation, general control nonderepressible 2 (GCN2) kinase senses intracellular amino acid levels and activates ATF4 for metabolic adaptation [9]. Consistently, ATF4′s transcriptional targets include those involved in antioxidant stress response, amino acid transportation and biosynthesis.

Both PERK and GCN2 directly phosphorylate and inactivate eukaryotic initiation factor 2α (eIF2α) [10]. The normal role of the eIF2 complex is to transport methionyl-tRNAs to the 40S ribosome. Specifically, this process requires the exchange of GDP for GTP within the complex, which is regulated by eIF2B. Phosphorylated eIF2α binds to eIF2B and inhibits the nucleotide exchange, and as a consequence, the rate of global translation is attenuated under these conditions. Interestingly, the translation of ATF4 is increased when the translational initiation is attenuated upon eIF2α phosphorylation [8]. Such regulatory effect is due to the unique 5′ UTR of ATF4 that is conserved across phyla. Specifically, the 5′ UTR of the ATF4 mRNA contains at least two upstream open reading frames (uORFs). Under normal conditions, ribosomes translate the first uORF (uORF1) and re-initiate translation of the next uORF (uORF2). Because ATF4 genes throughout phyla contain an uORF that overlaps with the ATF4 coding sequence, the main ATF4 ORF is almost never translated when the last uORF is efficiently recognized by the ribosome. However, when PERK or GCN2 are activated, the decrease of functional eIF2 complex makes the AUG start codon recognition less efficient. This allows the scanning ribosomes to occasionally bypass the final uORF and to give the ATF4 main ORF an opportunity to be recognized for translation [11]. Thus, the translation of ATF4 is enhanced in response to ER stress and starvation.

ATF4 is associated with a variety of diseases including Parkinson′s disease [12], congenital skeletal dysplasia [13], nephronophthisis [14]. In addition, recent studies show that ATF4 is involved in diverse cellular and physiologic processes ranging from the maintenance of hematopoietic stem-cell [15], glucose metabolism [16] to immune response [17]. The *Drosophila* genome has a conserved *Drosophila* ATF4 gene, which is also referred to as *cryptocephal (crc)*. Mutant *crc* larvae exhibit defects in molting and metamorphosis [18]. We had previously shown that, as in other organisms, ER stress induces *Drosophila* ATF4 expression [19]. Here, we report the development of a *Drosophila ATF4* reporter, in which a dsRed coding sequence placed under the control of the ATF4 5′ UTR. Using this reporter, we find that the expression of *Drosophila* ATF4 is also regulated by uORFs in the 5′UTR. In addition, chemically and genetically induced ER stress and starvation activates the ATF4 reporter *in vivo*. Moreover, we detect this reporter activity in a number of normally developing tissues, indicative of inherent stress. As ATF4 is known to have causal effects in the progression of various neurodegenerative or metabolic diseases, this tool may facilitate the study of ATF4 function in *Drosophila* models of human diseases.

## Materials and Methods

### Constructs

All *Drosophila* reporters were subcloned into *pCasper4* vector. To have the reporter transcripts expressed ubiquitously, the tubulin promoter and the SV40 3′ UTR were subcloned into NotI/KpnI and PstI sites, respectively. The 5′UTR sequence of *Drosophila* ATF4-RA was obtained through RT-PCR from *y*, *w* larvae, and the dsRed.T4 sequence was PCR amplified from the pPelican plasmid, respectively. The dsRed.T4 has much shorter maturation time compared to dsRed(RFP) (half-time<43 min), no significant green emission, and improved solubility [20]. The dsRed ORF (we designate dsRed.T4 as dsRed from this point forward) was fused 3′ of the ATF4 5′ UTR after three nucleotides (ACC), and the dsRed AUG codon matched the original position of the ATF4 start codon. This sequence was initially subcloned into pBluescript-SK(-), and subsequently moved to the *pCasper4* vector.

### 5′ RACE

Total RNA was extracted with TRIzol reagent (Ambion, USA) according to the manufacturer′s protocol. 5′ RACE was performed according to Invitrogen′s kit instructions (Invitrogen, USA). In brief, the first strand primer (GSP1) was annealed to the mRNA of 5′ ATF4-luc, mRNA was then copied into the cDNA with SUPERSCRIPTII RT. After labeling purified cDNA with dCTP and TdT, the dC-labeled cDNA was amplified using the abridged anchor primer (AAP) and GSP2 primer, and then the primary PCR product was amplified again using the abridged universal amplification primer (AUAP) and GSP3 primer. The secondary PCR products were analyzed by electrophoresis using a 1% agarose gel and extracted with a High Pure PCR Product Purification Kit (Roche). The extracted PCR product was cloned using a pLPS-B Blunt topo vector (ELPis biotech, Korea) and sequenced. The transcriptional start sites were determined as the first nucleotide that is 3′ to the adapter sequence ligated to the 5′ of the mRNA transcripts. The used primers were as follows: GSP1 primer 5′-ATTATAAATGTCGTTCG-3′, GSP2 primer 5′-CTGCAACTCCGATAAATAAC-3′, GSP3 primer 5′-GCATACGACGATTCTGTGAT-3′.

### Fly genetics

Genes were expressed in *Drosophila* through the standard Gal4/UAS system [21]. The following flies been described previously: *gmr-gal4*, *uas-Rh-1* ^*G69D*^ */Cyo* [19], *tub-gal4*, *uas-xbp1-EGFP/Cyo* [22], *uas-Aβ* [23], *uas-MJD-tr-Q78* [24]. UAS-lacZ line was obtained from Bloomington Stock Center.

### Immunohistochemistry

All fluorescent images were obtained with a Zeiss LSM710 confocal microscope, using a ×20 or x40 objective lens. The following antibodies were used: rabbit anti-dsRed antibody (1:50 from Clontech or 1:500 from Dr. S.W. Kang), rabbit anti-GFP (1:2000, Molecular Probes, catalogue no. A6455), mouse anti-armadillo (1:500; Developmental Studies Hybridoma Bank, N2 7A1, University of Iowa, USA), monoclonal anti-rhodopsin1 (1:500; Developmental Studies Hybridoma Bank, 4C5, University of Iowa, USA), actin-phalloidin (1:200; Molecular Probes). To generate the guinea-pig anti-ATF4 antibody, the full length of the ATF4 coding sequence was subcloned into XhoI and NotI sites in pET14b (Novagen). The resulting ~50 kDa His-tagged recombinant protein was purified to generate a polyclonal antibody. The antisera were subsequently affinity purified against the same epitope [19].

### Nutrient restriction

Hatched larvae were raised in apple-juice plates with active yeast paste at 25°C. Larvae were collected at 47~49 h After Egg Laying (AEL) and transferred to standard cornmeal food (5.9% w/v Glucose, 6.6% Cornmeal, 1.2% Baker's Yeast, 0.7% Agar in water) or to Nutrient Restriction (NR) medium (5% Sucrose, 1% Agar in PBS) for 18 h at 25°C.

### Feeding assay

Larvae were collected 47~49 h AEL. Larvae were starved for 4 h and then fed with 10 μg/mL tunicamycin, 1 μM thapsigargin, and 5 mM DTT in Schneider′s Drosophila medium (Gibco) for 5 h at 25°C. All chemicals were purchased from Sigma-Aldrich.

### RT-PCR

For cDNA synthesis, 1μg of RNA was transcribed using SuperScript First-Strand Synthesis Kit (Invitrogen, USA). PCR amplification was performed for 25 cycles using *taq* polymerase (Roche) according to manufacturer′s protocol. Primers used include the following: luciferase forward primer, 5′-CTCGCATGCCAGAGATCCTA-3′; luciferase reverse primer, 5′-AAGGCTCCTCAGAAACAGCT-3′; rp49 forward primer, 5′- AGATCGTGAAGAAGCGCACCAAG-3′; rp49 reverse primer, 5′- CACCAGGAACTTCTTGAATCCGG-3′. Rp49 was used as a housekeeping control to normalize the amounts of cDNA between each of the samples. Results were expressed as the relative expression of mRNA levels detected in control samples.

### Luciferase assay

The 5′UTR DNA of *Drosophila* ATF4-RA was obtained through RT-PCR from *y*, *w* larvae, subcloned into pGL3-basic vector (Promega, USA), and labeled as 5′ATF4-luc. The mutant constructs (5′ATF4.uORF1^AUA^-luc, 5′ATF4.uORF2^AUA^-luc, and 5′ATF4.uORF1^AUA^uORF2^AUA^-luc) were generated using the QuickChange site-directed mutagenesis kit (Stratagene). The sequence of the mutated DNA was verified by DNA sequencing. *Drosophila* S2 cells were transiently co-transfected with wild type or mutant reporter plasmid plus pRL (Renilla luc) using Effectene (Qiagen). After 96 h, luciferase activity was measured using DUAL-GIO luciferase assay system (Promega, USA). The firefly luciferase activity was normalized to Renilla luciferase activity.

## Result

### *Drosophila* ATF4 expression is regulated by a mechanism involving uORF

**Fig 1:**
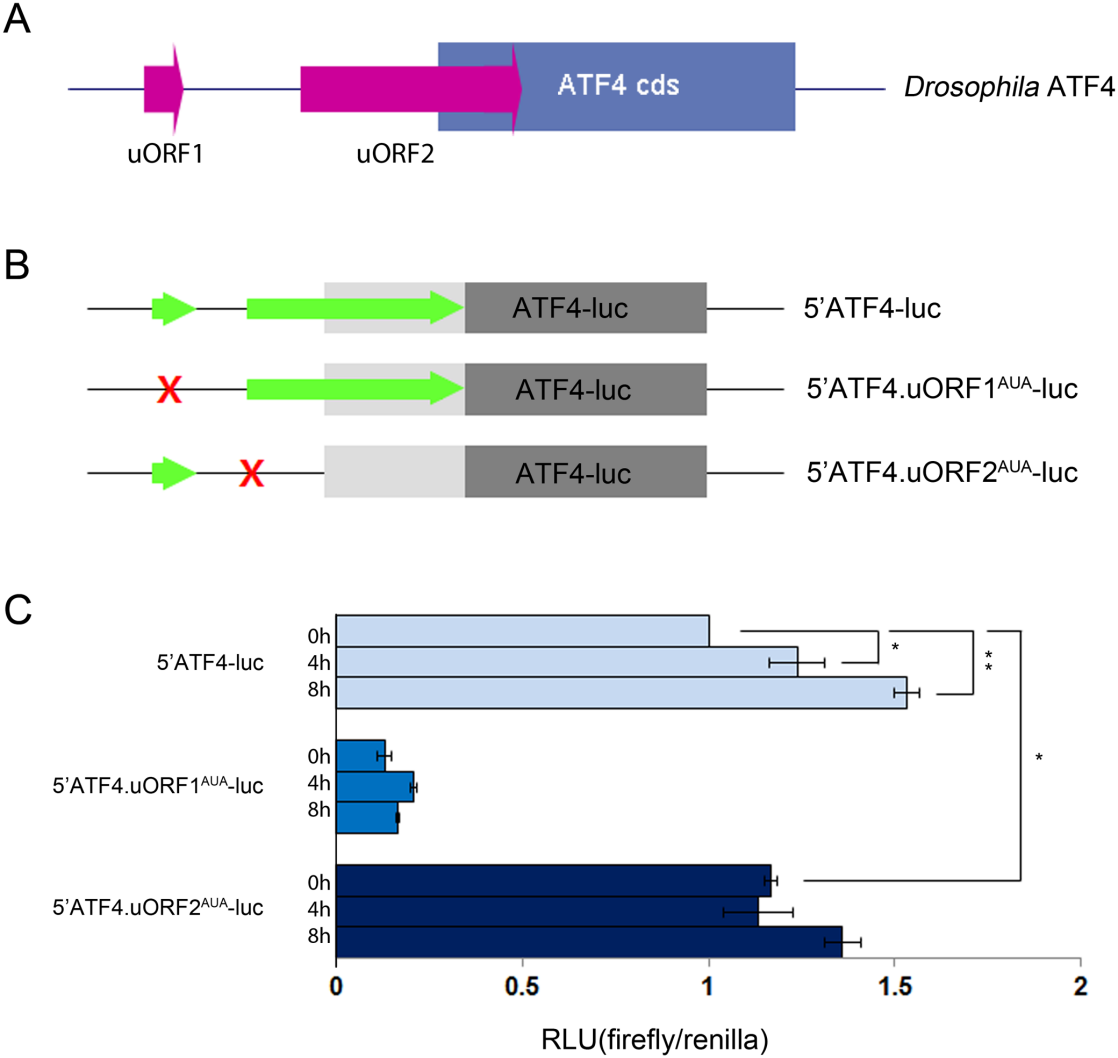
The uORFs of *Drosophila* ATF4 5′UTR mediate translation regulation. (A) Structure of the 5′ UTR of the *Drosophila* ATF4-RA mRNA. The two uORFs, such as uORF1 and uORF2, were present in the 5′ UTR. The uORF2 overlaps with the ATF4 ORF, but in a different reading frame. (B) The luciferase reporter construct used in this experiment. The green arrow represents the wild type version of uORF1 and uORF2, and the X indicates a mutation in the initiation codon at each uORF. (C) *Drosophila* S2 cells were transfected with the indicated ATF4-Luc plasmid and a control *Renilla* luciferase plasmid. The transfected cells were treated with 1mM of DTT for the indicated time points, 0, 4, and 8 h. Relative light units (RLU) indicates a ratio of firefly luciferase activity normalized with *Renilla* luciferase activity, and each value was derived from three independent transfections. Error bars show ± s.e.m. *p<0.05, **p<0.005.

In a previous study, we had shown that the level of *Drosophila* ATF4 protein increases in response to ER stress caused by misexpression of mutant Rhosopsin-1[19], which prompted us to examine whether the mechanism of ATF4 induction upon ER stress is conserved in *Drosophila*. In mammals, ATF4 expression is regulated in response to eIF2α phosphorylation by a mechanism involving two upstream open reading frames (uORFs) in 5′ UTR [11, 25]. As shown in Fig 1A, we found that *Drosophila* ATF4-RA (S1 Fig) also has two uORFs in its 5′UTR. To examine the role of these two uORFs in regulating ATF4 expression, we made a reporter construct with the tubulin alpha1 promoter driving the expression of a chimeric transcript containing the ATF4 5′UTR fused to the luciferase coding sequence. The construct was designed so as to determine the effect of the 5′ UTR on the translation of the luciferase reporter coding sequence. To assess the roles of the uORFs, we made additional constructs with uORF start codons mutated to AUA. The transcriptional start site of reporters was analyzed by 5′ RACE and sequencing, and the luciferase reporter has two uORFs in its 5′UTR (S2A Fig). We transfected these plasmids into cultured S2 cells and measured the luciferase activity driven by either the wild type 5′UTR, or those with uORF1^AUA^, or uORF2^AUA^ mutated 5′ UTR (Fig 1B). DTT treatment increased firefly luciferase activity in cells transfected with wild type luciferase reporter (5′ATF4-luc), which indicated that the ATF4 reporter translation is stimulated in response to ER stress. Mutation in uORF1 (5′ATF4.uORF1^AUA^-luc) resulted in a severe reduction in luciferase activity in S2 cells. However, when uORF2 was mutated, there was an increase in the luciferase activity, independent of ER stress (Fig 1C). In addition, when both of uORF1 and uORF2 were mutated, the effects of uORFs were abolished (S2B Fig). The levels of mRNA from the reporters were similar in all conditions (S2C Fig). These results are consistent with the idea that uORF1 and uORF2 have opposing roles in the translation of the main ORF, and that the translational regulatory mechanisms of ATF4 is conserved between *Drosophila* and mammals.

### Development of an *in vivo* ATF4 translational activation reporter

**Fig 2:**
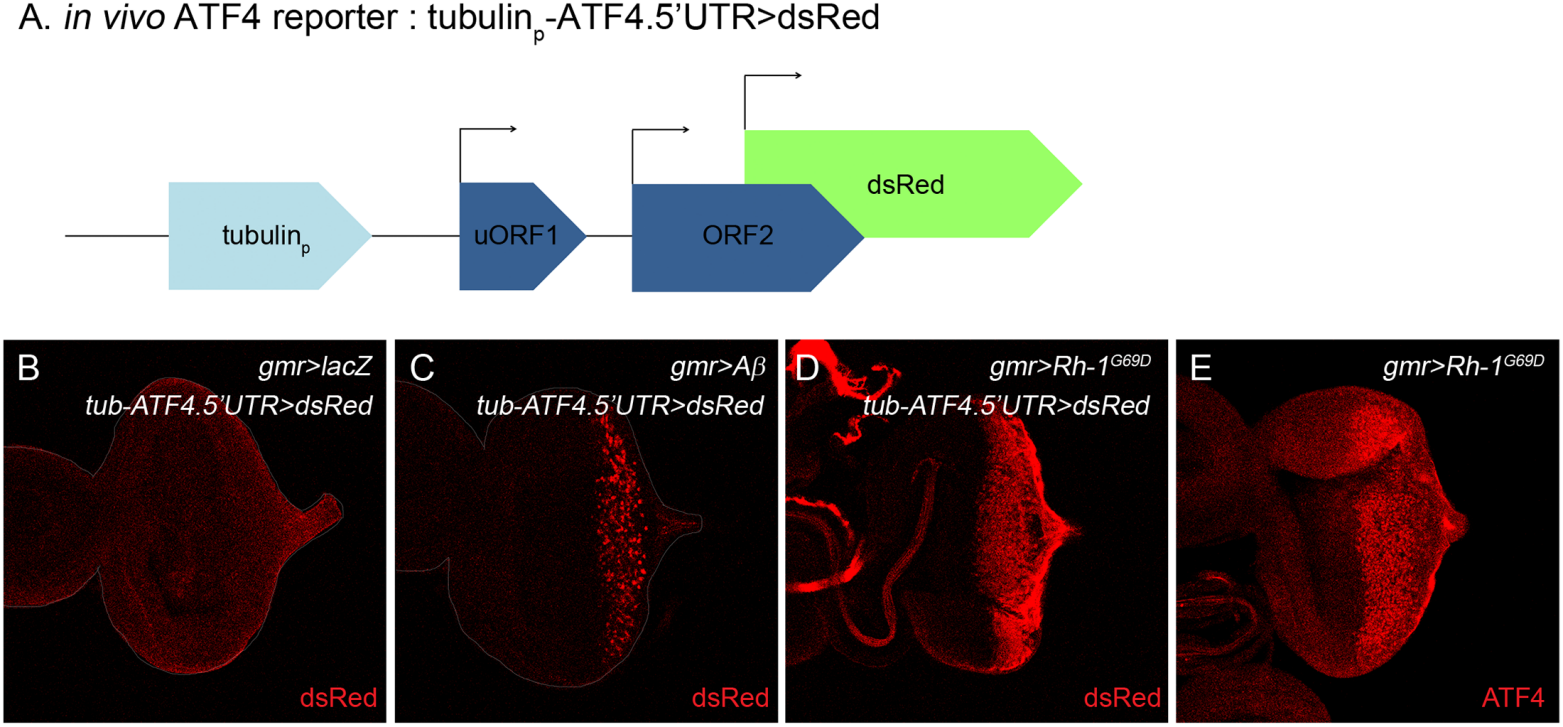
*In vivo* ATF4 reporter, *tub-ATF4*.*5′UTR>dsRed* responds to protein misfolding. (A) The structure of the ATF4 translational activation reporter, *tub-ATF4*.*5′UTR>dsRed*. The dsRed ORF replaces that of ATF4, thereby reporting ATF4′s translation. (B-E) Validation of the reporter response *in vivo*. Control eye discs did not show dsRed expression (B). Misexpression of Aβ activated the *tub-ATF4*.*5′UTR>dsRed* reporter, as evidenced by the expression of dsRed (C). The ATF4 reporter pattern in response to Rh-1^G69D^ misexpression in the eye disc (D). Endogenous ATF4 expression detected using anti-ATF4 antibody labeling (E).

To better understand how ATF4 expression is regulated during normal development as well as in ER stress, we developed an *in vivo* ATF4 reporter modified from ATF4 luciferase reporter. We placed a dsRed gene under the control of the ATF4 5′UTR (Fig 2A). To determine whether the *in vivo* ATF4 reporter responds to ER stress, we misexpresssed a mutant Rhodopsin-1(Rh-1^G69D^) in larval eye imaginal discs through the *gmr-gal4* driver. Under these conditions, we observed dsRed reporter induction in larval eye discs that were expressing Rh-1^G69D^ (Fig 2D). Although the ATF4 antibody staining was not sensitive enough to detect the endogenous ATF4 expression in normally developing tissues, we were able to detect the endogenous ATF4 protein induction by Rh-1^G69D^ misexpression (Fig 2E), which was nearly identical to the pattern of the dsRed reporter induction. Next, we tested if other aggregation prone proteins can activate the ATF4 translational reporter. Among those tested were MJD-tr-Q78 [24] and Aβ [23]. MJDtr-Q78 expression through the *gmr-gal4* driver in larval eye imaginal discs caused a severe eye ablation phenotype (S3C Fig), but it did not activate detectable levels of the ATF4 reporter (data not shown). By contrast, when we co-expressed the ATF4 reporter together with Aβ, a peptide that underlies Alzheimer′s disease, we observed dsRed induction, albeit at a reduced level (Fig 2C). These results suggest that the newly developed *in vivo* ATF4 reporter responds to some stress-imposing proteins in vivo.

### ER stress activates the ATF4 expression reporter *in vivo*

**Fig 3:**
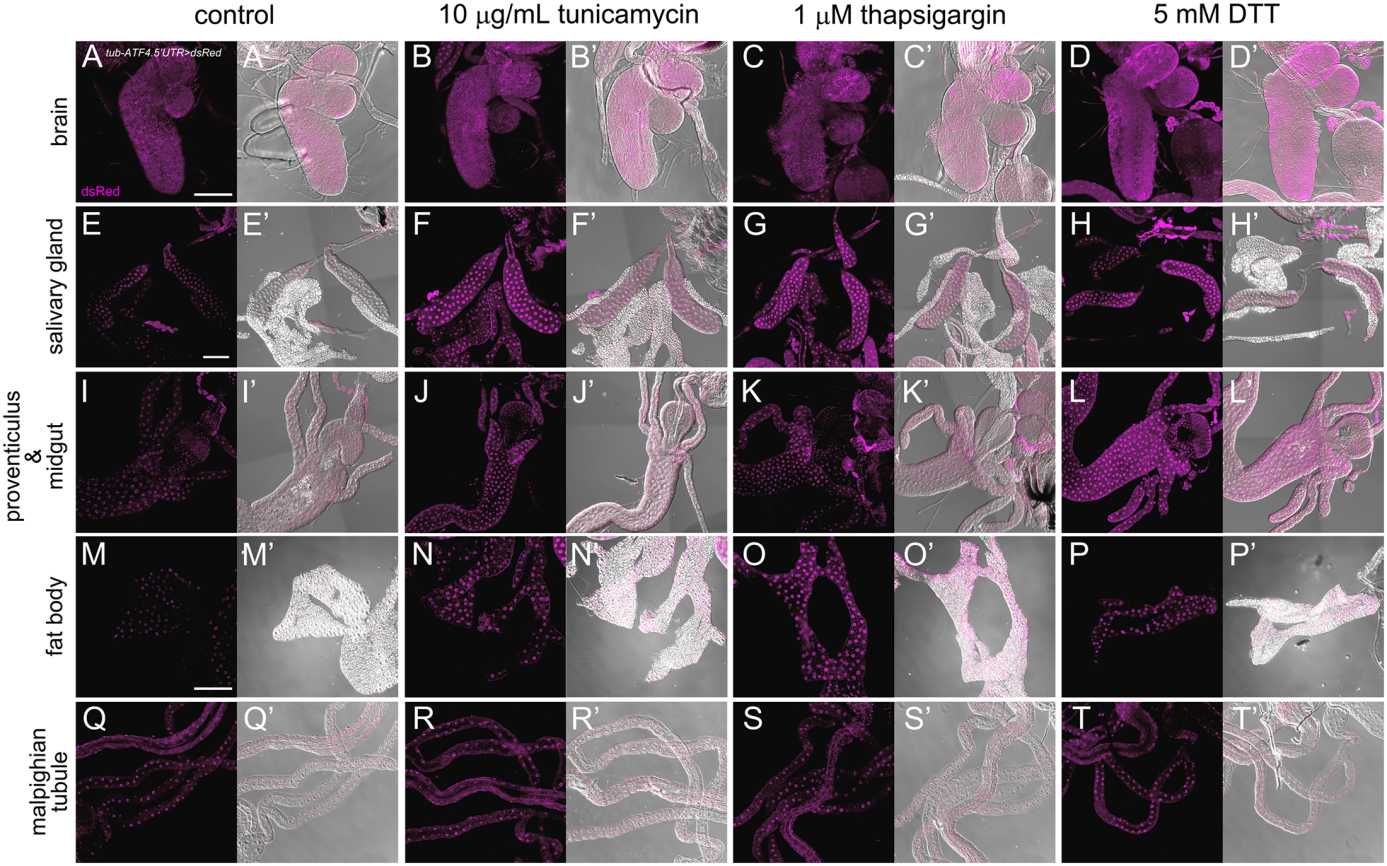
The expression of *ATF4*.*5′UTR>dsRed* upon ER stress. 2^nd^ instar larvae expressing ATF4 reporter were starved for 4 h and then fed with control (A, E, I, M, Q) or 10 μg/mL tunicamycin (B, F, J, N, R) or 1 μM thapsigargin (C, G, K, O, S) or 5 mM DTT (D, H, L, P, T) in Schneider′s *Drosophila* medium for 5 h. Next, whole mount labeling of tissues were performed with the anti-dsRed antibody. DsRed expressions in salivary gland (E-H), gut (I-L) and fat body (M-P) increased in response to ER stress-inducing agents. In brain (A-D) and mapighian tubule (Q-T), feeding with ER stress-inducing agents did not noticeably alter dsRed expression. The scale bars in (A, E, M) represent 100 μm.

While the *tub-ATF4*.*5′UTR>dsRed* remained inactive in unstressed larval eye discs, we found that this reporter activity was detected in various tissues including the brain, salivary glands, proventiculus, midgut, fat body, and malpighian tubules during normal feeding (Fig 3A, 3E, 3I, 3M and 3Q), suggestive of inherent stress during normal development. To independently characterize the translational profile of ATF4 in response to ER stress *in vivo*, we fed 2^nd^ instar larvae with ER stress causing chemicals. After feeding with 10 μg/mL tunicamycin, 1 μM thapsigargin and 5 mM DTT for 5 h, larvae were dissected and stained with anti-dsRed antibody. Under these conditions, expression of *tub-ATF4*.*5′UTR>dsRed* increased broadly. Especially, there was a marked induction of *tub-ATF4*.*5′UTR>dsRed* in the salivary gland (Fig 3E–3H). In addition, increased levels of *tub-ATF4*.*5′UTR>dsRed* were observed in the proventiculus, midgut and fat body upon rearing larvae with ER stress causing chemicals (Fig 3I–3P). However, there were no noticeable changes of dsRed expression in the larval brain and malpighian tubules (Fig 3A–3D and 3Q–3T). The *in vivo* ATF4 reporter was further validated in *Drosophila* S2 cells. This reporter also responded to the ER stress causing chemical, DTT, tunicamycin, thapsigargin in *Drosophila* S2 cells (S4 Fig). These results indicate that the ATF4 reporter can detect both stress caused by inherent and exogenous sources, and such response is tissue dependent.

### The *in vivo* ATF4 reporter responds to nutritional deprivation

**Fig 4:**
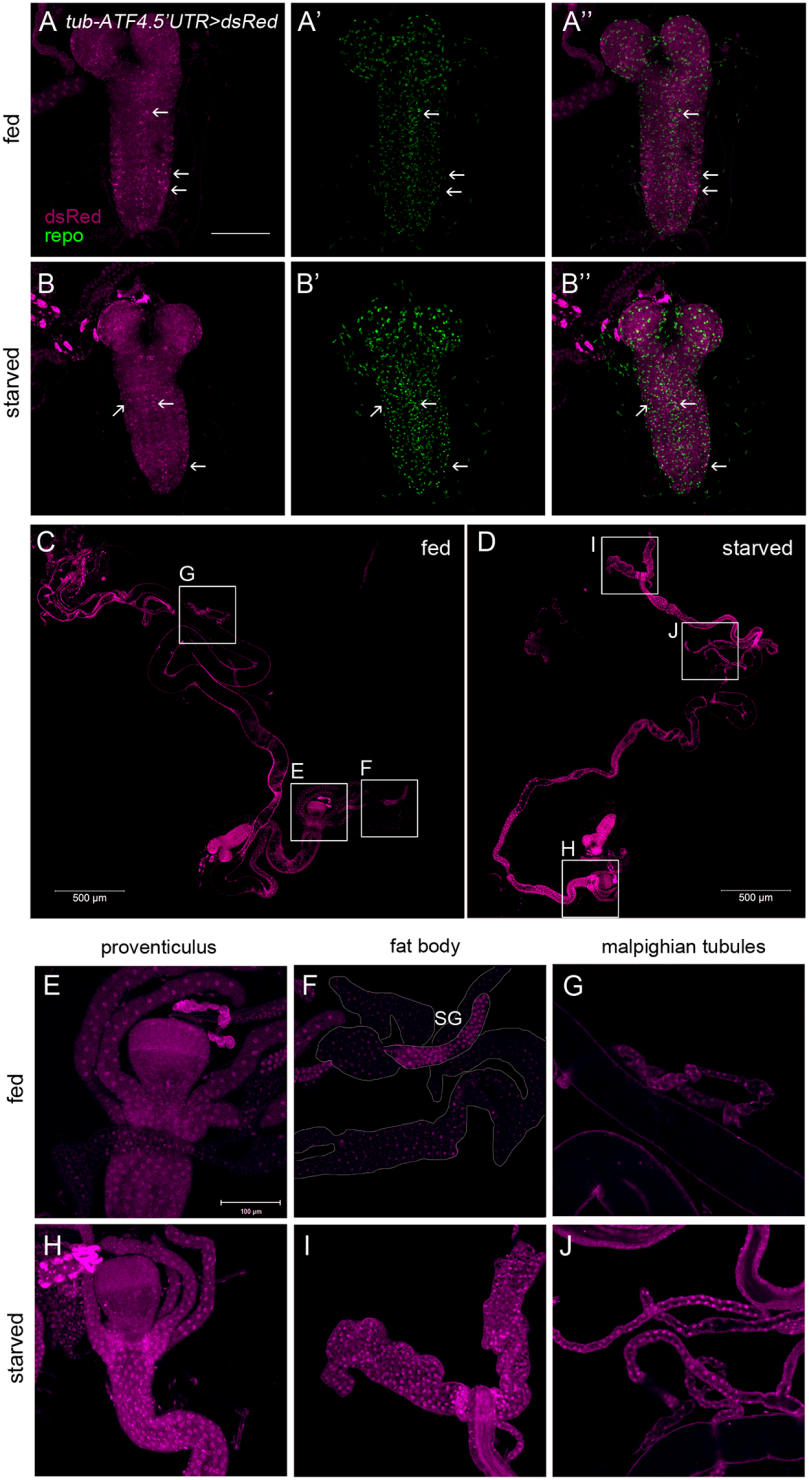
*tub-ATF4*.*5′UTR>dsRed* expression is activated in response to nutritional deprivation. (A, B) *tub-ATF4*.*5′UTR>dsRed* reporter in the larval brain. dsRed was observed in the brain (white arrowhead). However, the expression was not significantly changed in larvae reared in protein deficient food. dsRed expression did not co-localize with anti-repo labeling, which marks glial cells (green). (C, D) dsRed expression in larval tissues expressing *tub-ATF4*.*5′UTR>dsRed* reporter. The *in vivo* ATF4 reporter expression was enhanced in response to nutritional restriction for 18 h in 2^nd^ instar larvae. Specifically, gut and fat body showed high levels of dsRed. (E-J) show higher magnification images of the inset in (C, D). The scale bar in (A) and (E) represents 100 μm for (A, B and E-J) and that in C, D represents 500 μm.

As introduced earlier, ATF4 translation is activated in response to a number of other stress conditions, including amino acid deprivation [25, 26]. To test whether our *in vivo* ATF4 reporter is activated in response to nutrient restriction, 2^nd^ instar larvae were reared in either standard food or in a nutritionally restricted condition (devoid of amino acids) for 18 h. In the standard food, we found a reproducible pattern of dsRed expression in the optic lobe and brain stem. We performed double labeling with the anti-repo (glial marker) antibody, which did not co-localize with the ATF4 reporter (Fig 4A′′). Although the *tub-ATF4*.*5′UTR>dsRed* reporter was active in the brain, there were no significant changes of the induction of dsRed between standard food and restricted food (Fig 4A and 4B). However, *tub-ATF4*.*5′UTR>dsRed* reporter was activated at high levels in the intestine when larvae were reared in restricted food, when compared with standard food (Fig 4C and 4D). Also in the larva, dsRed expression was prominently induced in the proventiculus, fat body, and malpighian tubules (Fig 4E–4J). As ATF4 is just one of the three transcription factors that mediate the UPR, we examined whether the other UPR pathways also respond to nutritional deprivation. We specifically tested a pathway mediated by an ER stress activated RNase, IRE1, which triggers the mRNA splicing of the transcription factor, xbp1. The activation of this pathway can be detected through the reporter, xbp1-EGFP, in which EGFP is expressed in frame only when ER-stress triggers the splicing of the xbp1 mRNA [27]. As shown in S5 Fig, EGFP expression did not change in noticeably upon nutrient restriction. These results indicate that ATF4 is specifically activated by nutrient restriction, suggesting that ATF4 may have an essential role in energy metabolism.

### *In vivo* ATF4 reporter is active in photoreceptors of *Drosophila*

**Fig 5:**
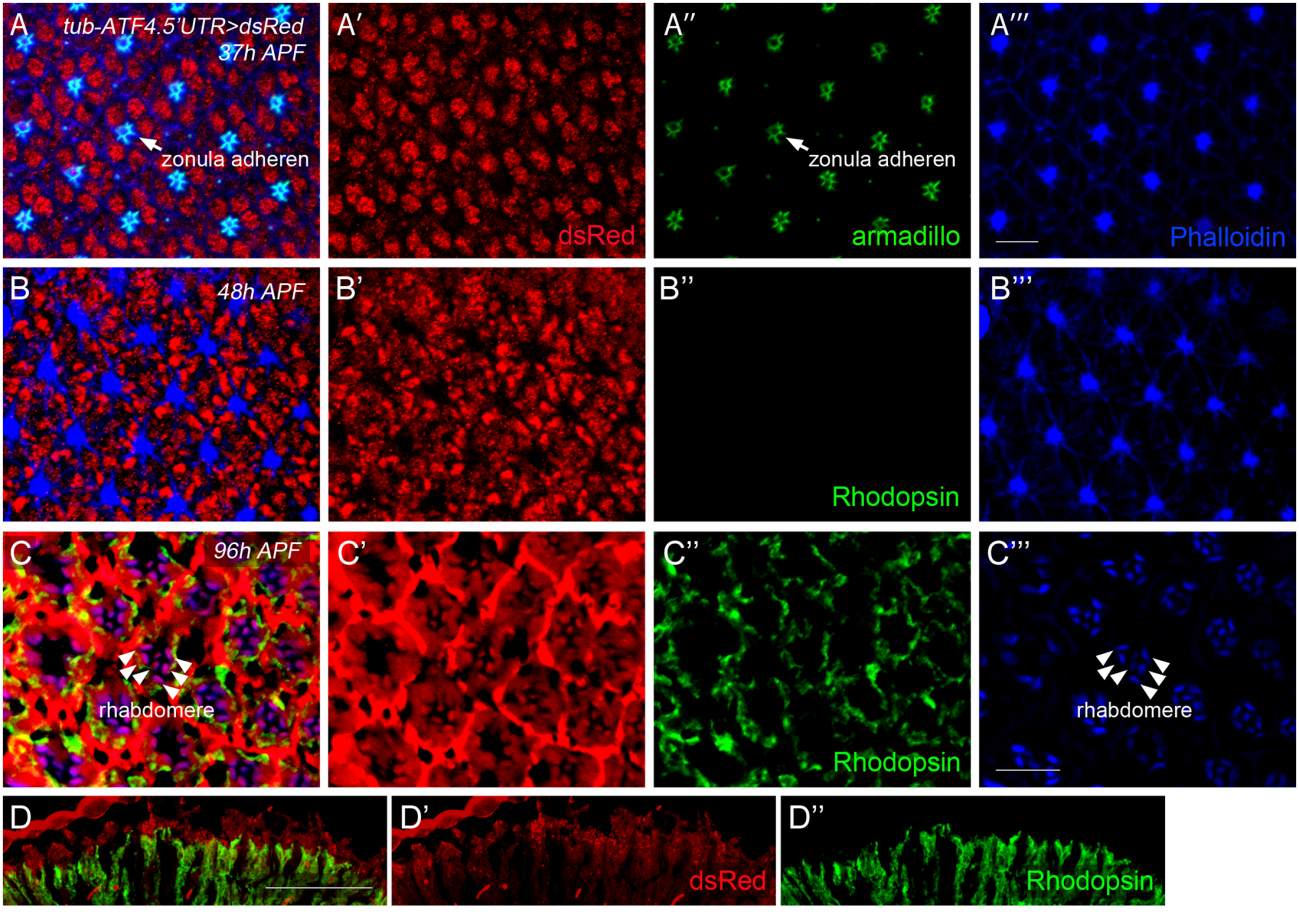
*In vivo* ATF4 reporter is activated in the photoreceptor cells. (A) At 37% pupal development (37h APF), retina were stained with anti-dsRed (red), anti-armadillo (arrowhead, marker for zonula adherens, green), and anti-actin-phalloidin (blue), respectively. DsRed is expressed at this stage. (B) At 48% of pupal development (48h APF), ATF4 reporter was still active, but, rhodopsin was not detected at this stage. (C) At 96% pupal development retina (96h APF), ATF4 reporter activity was detected at the photoreceptor cells. (D) The expression of dsRed in the adult fly retina, but not in the photoreceptor. Red indicates ATF4 reporter and green is rhodopsin staining. The scale bar in (A′′′) represents 5 μm for (A-C) and that in D represents 50 μm.

A previous study had shown that the UPR sensor, xbp1-EGFP is active in photoreceptors during the second half of pupal development [28]. To determine whether the ATF4 branch of signaling is similarly activated in photoreceptor cells, we examined the ATF4 reporter activity during pupal and adult stages. In the early stage of pupa (37 h After puparium formation: APF), we were able to detect the ATF4 reporter activity in photoreceptor cells (Fig 5A and 5A′). This reporter activity remained at 48 h APF, but the pattern of expression was altered (Fig 5B and 5B′). At 96 h of puparium, dsRed was still expressed in photoreceptor cells. In adult retina, we found that dsRed reporter was expressed, but not within the photoreceptor (Fig 5D–5D′′), similar to the reported xbp1-EGFP pattern that overlapped with Homothorax-positive lattice cells [28]. These results indicate that ATF4 translation is stimulated during normal photoreceptor development.

### The ATF4 reporter is highly expressed in the adult male reproductive organs

**Fig 6:**
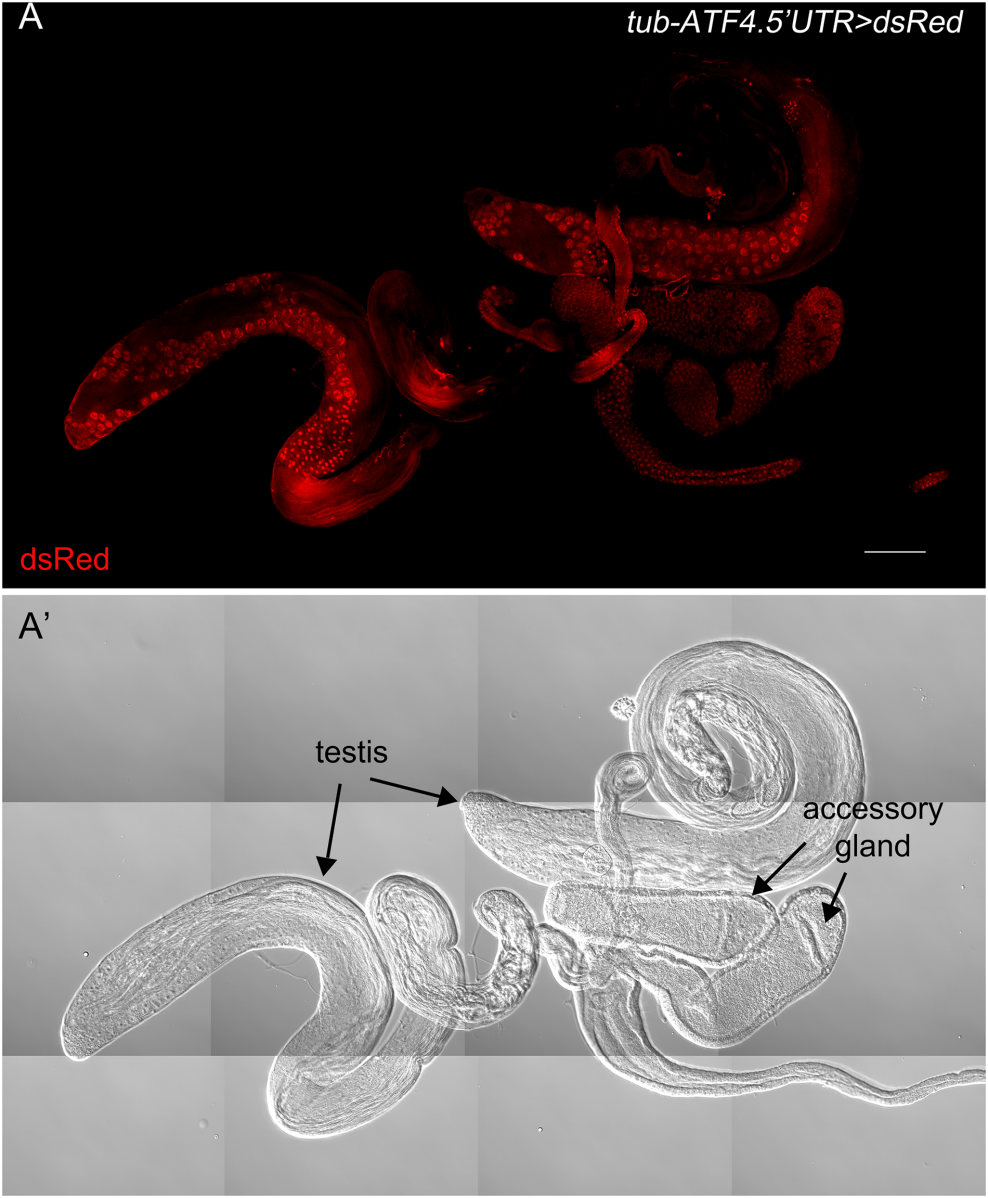
*tub-ATF4*.*5′UTR>dsRed* expression in the male reproductive organ. Dissected male reproductive tissue is stained with anti-dsRed antibody. DsRed signal is shown in the testis, accessory gland, and the ejaculatory duct. The scale bar in (A) represents 100 μm.

Previous studies on the xbp1 reporters, xbp1-EGFP and xbp1_p_>dsRed in *Drosophila*, indicated that the *xbp1* pathway of the UPR is active within the adult male reproductive organs, but not in females [22, 27]. Specifically, xbp1-EGFP reporter was detected in the accessory glands and a limited area of the testis [22]. On the other hand, xbp1_p_>dsRed was expressed in the accessory glands and the ejaculatory duct, but not in the testis itself [27]. To test if the ATF4 reporter is active in the male reproductive organ, we dissected the adult reproductive organ and stained with anti-dsRed antibody. DsRed was observed not only in the accessory glands and the ejaculatory duct, but also in a limited area of the testicular duct and the testis (Fig 6). This result is consistent with the idea that UPR signaling is active in the organ having high protein secretory activity.

## Discussion

Here, we report the development of an *in vivo* ATF4 reporter, *tub-ATF4*.*5′UTR>dsRed*, designed to show the translational activation pattern of ATF4. In the developing fly, it has been reported that the UPR pathways are active in healthy tissues with high protein secretion load [22, 27]. Previously generated ATF4 antibody failed to detect the endogenous ATF4 expression in normally developing tissues, but our newly developed ATF4 reporter marked stressed cells during development, and the activation pattern was similar to that of the other established UPR reporters, xbp1p>dsRed and xbp1-EGFP [27, 29]. Among those tissues with positive signals were the salivary gland, gut, and the male reproductive organ. However, unlike the xbp1 reporter, we found the ATF4 reporter activity in a restricted region of testis (Fig 6). As *Atf4*-/- male mice are infertile, and spermatogenesis is disrupted in the *Atf4-/-* testis [30, 31], these results suggest that *Drosophila* ATF4 may have an evolutionarily conserved role in spermatid differentiation. Previous studies reported that UPR controls the stem cell maintenance [15, 32], and therefore, it is possible that ATF4 may govern the proliferation of stem cells in the testis. In addition, *tub-ATF4*.*5′UTR>dsRed* reporter was activated in the pupal retina. While the xbp1-EGFP reporter was transiently activated in the mid and late stages of pupa retina, the ATF4 reporter expression was observed in the early stage of pupa (37 h APF) and maintained until adulthood. *Drosophila* Rh1 is detectable at 78 h APF, which is localized to the cell bodies and stalk [33]. At 80–82 APF, Rh1 is first trafficked to the rhabdomeres, and then shifts to the base of rhabdomeres at 96 APF, which is identical with Fig 5C. As Rh1 is a membrane protein, which requires chaperones to acquire its correct conformation, we believe that ATF4 is expressed earlier than Rh1 to help the induction of chaperone in the retina to fold Rh1 properly. As ATF4 has an essential role in the eye development in mouse [34], and perhaps, *Drosophila* ATF4 plays an analogous role. The ATF4 reporter also responded to nutritional deprivation. As shown in Fig 4, *tub-ATF4*.*5′UTR>dsRed* reporter was distinctly observed in gut, fat body and malpighian tubules under these conditions. As these organs sense nutrients and modulate metabolism [35, 36], ATF4 may be involved in this process by controlling the expression of essential genes sensitive to nutritional status.

Notably, our *tub-ATF4*.*5′UTR>dsRed* reporter was activated in response to disease causing gene expression, including Rh-1^G69D^ and Abeta. In conclusion, we believe that this reporter may be used to explore possible involvements of ATF4 in *Drosophila* development, stress response and nutritional deprivation.

## Supporting Information

S1 Fig Sequence of the 5′-leader of *Drosophila ATF4 (crc-RA)* mRNA. Blue letters indicate the translational regions of uORF1. Red letters indicate the translational regions of uORF2. The start codon is indicated by bold letters. Underlines indicate the stop codons. uORF1 and uORF2 have a start codon and a stop codon, respectively. Dark blue letters indicate the start codon of *ATF4*. uORF2 overlaps 125 nt of the *ATF4*-coding region.
(TIF)

S2 Fig The two uORFs present in 5′UTR of the *Drosophila* ATF4 mRNA regulate the translation. (A) 5′-RACE was carried out for ATF4-Luc using RNA prepared from *Drosophila* S2 cells expressing the ATF4-luciferase reporters. (top panel) 5′-RACE products were separated and visualized by electrophoresis using a 1% agarose gel, with markers of the indicated size in base pairs represented on the right. (bottom panel) The multiple sequence alignment of ATF4-RA 5′UTR and 5′RACE results of ATF4-luciferase reporters. The start codons of uORF1 and uORF2 in the analysis of ATF4 translational control are indicated with the black box. The arrow indicates the transcription start site of the ATF4-luciferase reporters. The translation start site of luciferase is shown in purple letters. The alignments were conducted using the MultAlin Multiple sequence alignment tool (http://multalin.toulouse.inra.fr/multalin/). (B) *Drosophila* S2 cells were transfected with the indicated ATF4-Luc plasmids and a control *Renilla* luciferase plasmid. The transfected cells were treated with 0.5 μM of Tg for 0, 4, and 8 h. RLU indicates a ratio of firefly luciferase activity normalized with *Renilla* luciferase activity. (C) *Drosophila* S2 cells were transfected with the indicated ATF4-Luc plasmid and treated with 1 mM DTT for 0, 4 and 8 h. (top panel) Levels of luciferase mRNAs were monitored by RT-PCR analysis. (bottom panel) Each bar represents the ratio of luciferase mRNA to that of rp49 mRNA. Data are expressed as the mean ± SEM. Gel images are representative of three independent experiments. PCR band intensities were measured with Image J.
(TIF)

S3 Fig External adult eyes. A control adult eye with wild-type morphology is shown in (A), Aβ expressing fly (B), MJD-tr-Q78 expressing fly (C).
(TIF)

S4 Fig *Tub-ATF4*.*5′UTR>dsRed* reporter is activated by various stresses. *Drosophila* S2 cells transfected with *tub-ATF4*.*5′UTR>dsRed* were incubateded with the ER-stress causing chemicals, DTT (1 mM), tunicamycin (Tu;10 μg/ml), thapsigargin (Tg;1 μM) in S2 medium or grown in a culture media lacking amino acids for 8 h. The upper panel shows anti-dsRed westerns to detect *tub-ATF4*.*5′UTR>dsRed* reporter activation, whereas the lower panel show anti-Profilin blots as a loading control. Negative control: untransfected cells; NT:non-treated cells.
(TIF)

S5 Fig Xbp1 splicing reporter, xbp1-EGFP does not respond to nutritional deprivation. UAS-xb1-EGFP is expressed under the control of the tubulin-gal4 driver. The 2^nd^ instar larvae were grown in normal food (A) or in 5% sucrose food that is devoid of amino acids (B) for 18 hours, dissected, and stained with anti-GFP antibody. The level of GFP did not change significantly. GFP staining (green) indicates xbp1 splicing, and red is repo staining. The scale bar in (A′ and B′) represents 200 μm.
(TIF)
