# Supplementary figures and images for: A Drosophila Reporter for the Translational Activation of ATF4 Marks Stressed Cells during Development
Source: PLoS One. 2015 May 15;10(5):e0126795. doi: 10.1371/journal.pone.0126795 (PMC4433282; doi:10.1371/journal.pone.0126795)

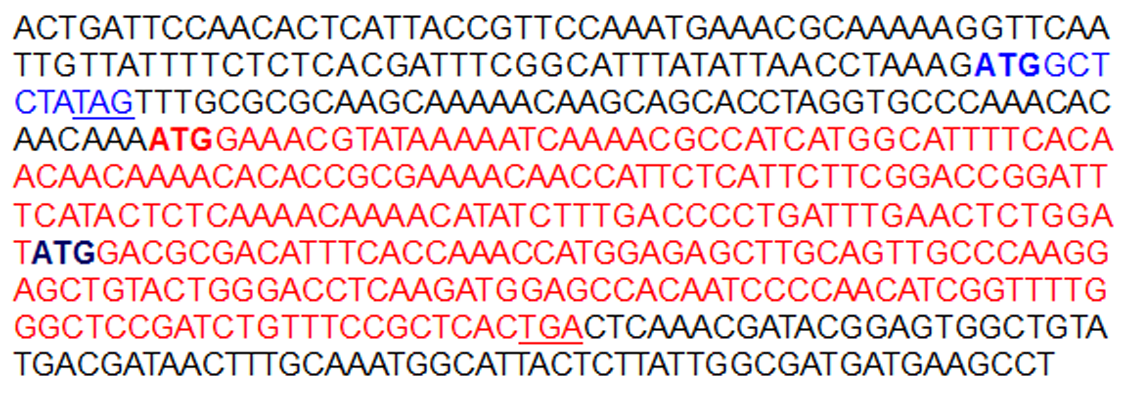

Supplement: S1 Fig — Blue letters indicate the translational regions of uORF1. Red letters indicate the translational regions of uORF2. The start codon is indicated by bold letters. Underlines indicate the stop codons. uORF1 and uORF2 have a start codon and a stop codon, respectively. Dark blue letters indicate the start codon of ATF4. uORF2 overlaps 125 nt of the ATF4-coding region. (TIF) [file pone.0126795.s001.tif]

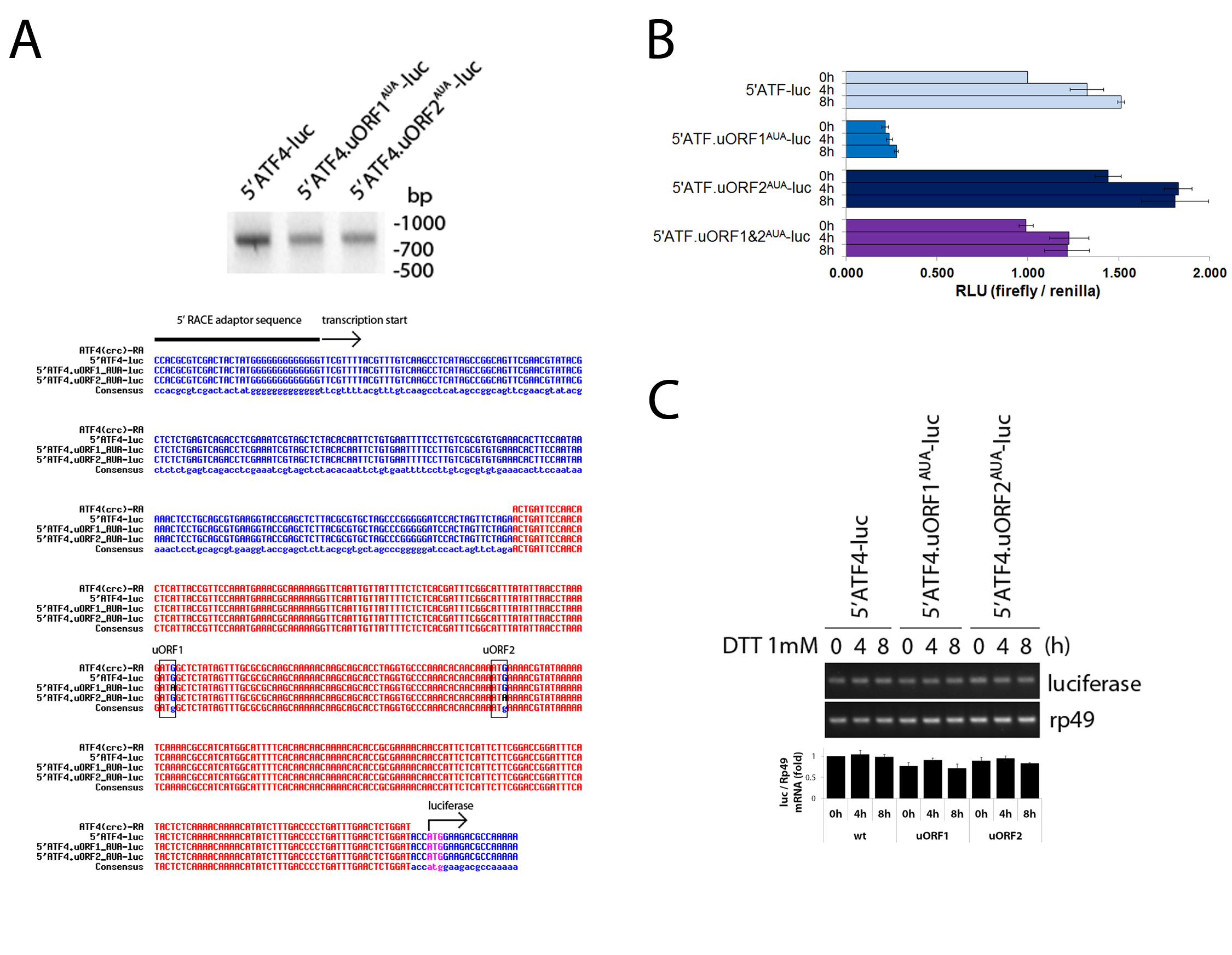

Supplement: S2 Fig — (A) 5′-RACE was carried out for ATF4-Luc using RNA prepared from Drosophila S2 cells expressing the ATF4-luciferase reporters. (top panel) 5′-RACE products were separated and visualized by electrophoresis using a 1% agarose gel, with markers of the indicated size in base pairs represented on the right. (bottom panel) The multiple sequence alignment of ATF4-RA 5′UTR and 5′RACE results of ATF4-luciferase reporters. The start codons of uORF1 and uORF2 in the analysis of ATF4 translational control are indicated with the black box. The arrow indicates the transcription start site of the ATF4-luciferase reporters. The translation start site of luciferase is shown in purple letters. The alignments were conducted using the MultAlin Multiple sequence alignment tool (http://multalin.toulouse.inra.fr/multalin/). (B) Drosophila S2 cells were transfected with the indicated ATF4-Luc plasmids and a control Renilla luciferase plasmid. The transfected cells were treated with 0.5 μM of Tg for 0, 4, and 8 h. RLU indicates a ratio of firefly luciferase activity normalized with Renilla luciferase activity. (C) Drosophila S2 cells were transfected with the indicated ATF4-Luc plasmid and treated with 1 mM DTT for 0, 4 and 8 h. (top panel) Levels of luciferase mRNAs were monitored by RT-PCR analysis. (bottom panel) Each bar represents the ratio of luciferase mRNA to that of rp49 mRNA. Data are expressed as the mean ± SEM. Gel images are representative of three independent experiments. PCR band intensities were measured with Image J. (TIF) [file pone.0126795.s002.tif]

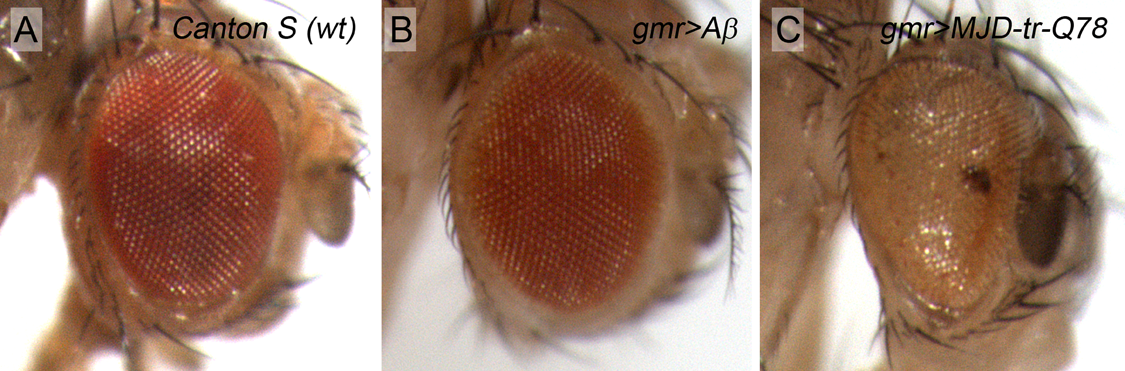

Supplement: S3 Fig — A control adult eye with wild-type morphology is shown in (A), Aβ expressing fly (B), MJD-tr-Q78 expressing fly (C). (TIF) [file pone.0126795.s003.tif]

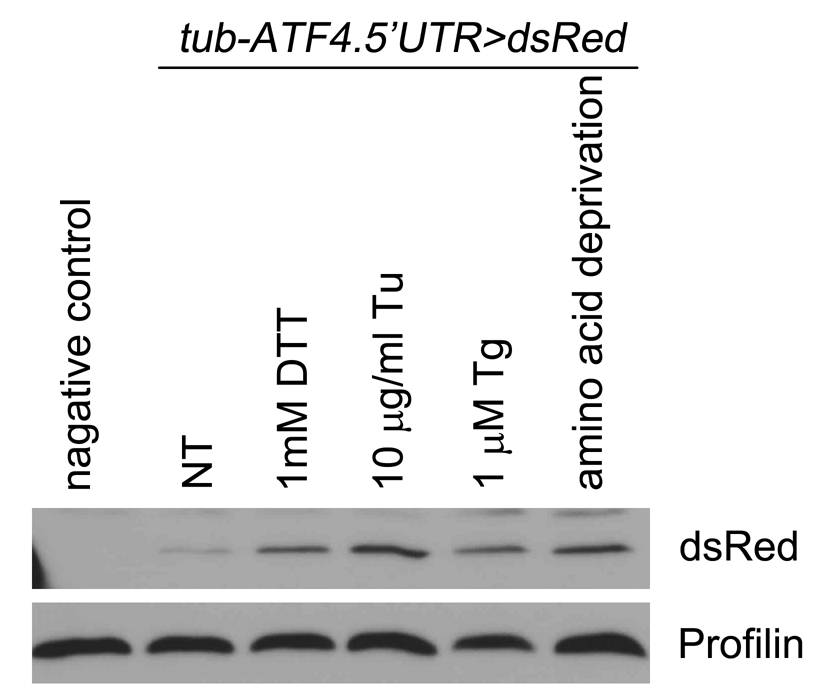

Supplement: S4 Fig — Drosophila S2 cells transfected with tub-ATF4.5′UTR>dsRed were incubateded with the ER-stress causing chemicals, DTT (1 mM), tunicamycin (Tu;10 μg/ml), thapsigargin (Tg;1 μM) in S2 medium or grown in a culture media lacking amino acids for 8 h. The upper panel shows anti-dsRed westerns to detect tub-ATF4.5′UTR>dsRed reporter activation, whereas the lower panel show anti-Profilin blots as a loading control. Negative control: untransfected cells; NT:non-treated cells. (TIF) [file pone.0126795.s004.tif]

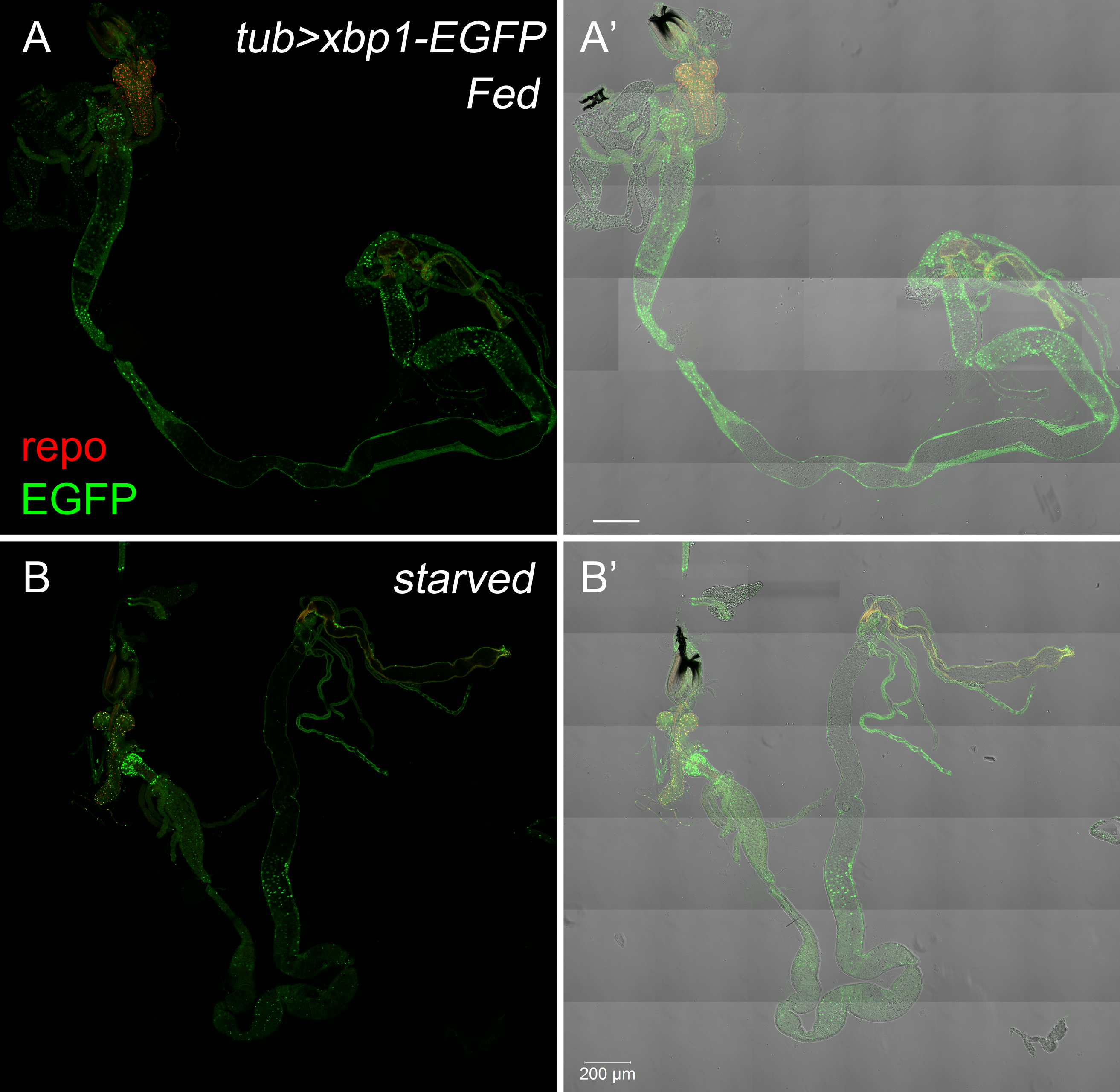

Supplement: S5 Fig — UAS-xb1-EGFP is expressed under the control of the tubulin-gal4 driver. The 2nd instar larvae were grown in normal food (A) or in 5% sucrose food that is devoid of amino acids (B) for 18 hours, dissected, and stained with anti-GFP antibody. The level of GFP did not change significantly. GFP staining (green) indicates xbp1 splicing, and red is repo staining. The scale bar in (A′ and B′) represents 200 μm. (TIF) [file pone.0126795.s005.tif]
